# Three-dimensional growth as multicellular spheroid activates the proangiogenic phenotype of colorectal carcinoma cells via LFA-1-dependent VEGF: implications on hepatic micrometastasis

**DOI:** 10.1186/1479-5876-6-57

**Published:** 2008-10-09

**Authors:** María Valcárcel, Beatriz Arteta, Arrate Jaureguibeitia, Aritz Lopategi, Iñigo Martínez, Lorea Mendoza, Francisco J Muruzabal, Clarisa Salado, Fernando Vidal-Vanaclocha

**Affiliations:** 1 Pharmakine Ltd., Bizkaia Technology Park, Derio, Bizkaia-48160, Spain; 2 Basque Country University School of Medicine & Dentistry, Dept. Cell Biology and Histology, Bizkaia-48940, Spain; 3 Fernando Vidal-Vanaclocha, Department of Cellular Biology and Histology, School of Medicine and Dentistry, University of the Basque Country, Leioa, Bizkaia-48940, Spain

## Abstract

**Background:**

The recruitment of vascular stromal and endothelial cells is an early event occurring during cancer cell growth at premetastatic niches, but how the microenvironment created by the initial three-dimensional (3D) growth of cancer cells affects their angiogenesis-stimulating potential is unclear.

**Methods:**

The proangiogenic profile of CT26 murine colorectal carcinoma cells was studied in seven-day cultured 3D-spheroids of <300 μm in diameter, produced by the hanging-drop method to mimic the microenvironment of avascular micrometastases prior to hypoxia occurrence.

**Results:**

Spheroid-derived CT26 cells increased vascular endothelial growth factor (VEGF) secretion by 70%, which in turn increased the *in vitro *migration of primary cultured hepatic sinusoidal endothelium (HSE) cells by 2-fold. More importantly, spheroid-derived CT26 cells increased lymphocyte function associated antigen (LFA)-1-expressing cell fraction by 3-fold; and soluble intercellular adhesion molecule (ICAM)-1, given to spheroid-cultured CT26 cells, further increased VEGF secretion by 90%, via cyclooxygenase (COX)-2-dependent mechanism. Consistent with these findings, CT26 cancer cells significantly increased LFA-1 expression in non-hypoxic avascular micrometastases at their earliest inception within hepatic lobules *in vivo*; and angiogenesis also markedly increased in both subcutaneous tumors and hepatic metastases produced by spheroid-derived CT26 cells.

**Conclusion:**

3D-growth *per se *enriched the proangiogenic phenotype of cancer cells growing as multicellular spheroids or as subclinical hepatic micrometastases. The contribution of integrin LFA-1 to VEGF secretion via COX-2 was a micro environmental-related mechanism leading to the pro-angiogenic activation of soluble ICAM-1-activated colorectal carcinoma cells. This mechanism may represent a new target for specific therapeutic strategies designed to block colorectal cancer cell growth at a subclinical micrometastatic stage within the liver.

## Background

During the earliest stages of the hepatic metastasis process, microvascular arrest and residency of disseminated cancer cells results in the generation of small subclinical foci of reversible characteristics at liver premetastatic niches [1]. At this avascular stage, single cancer cells become multicellular foci. In turn, this demands a functional adaptation of clonogenic cancer cells to the new microenvironment created by their own three-dimensional (3D) tissue organization, where ambient pressure and metabolic substrate concentration changes are occurring [2].

Using an experimental hepatic metastasis model [3], we reported the angiogenesis-stimulating potential activation in avascular micrometastases prior to hypoxia occurrence, leading to the intratumoral recruitment of vasculature-committed stromal cells [3]. This pre-angiogenic event is connected to hepatic micrometastasis development, but how the 3D status of cancer cell growth *per se *contributes to angiogenic-stimulating potential upregulation in non-hypoxic micrometastases is unclear.

Spheroids represent a popular *in vitro *3D tissue structure that mimics *in vivo *tumor tissue organization and microenvironment [4,5]. Within the spheroid, spatial cancer cell arrangements and tissue-like features are constituted that can recapitulate the architecture of the original tumor [6,7]. Metabolic and signal gradients, 3D-based cell-cell interactions and communication, and position coordinate-dependent proliferation and gene/protein expression patterns are also established [5,8,9] which can even affect the expression of important cell adhesion molecules [10].

Because a complex tissue-reconstitution program evolves during compact cancer cell growth *in vivo*, we hypothesized that angiogenic-stimulating factor production may be upregulated during *in vitro *3D-growth of cancer cells, even prior to hypoxia occurrence. However, how this is regulated, which biomarkers are defining the process, and which functional significance it has *in vivo *are unclear questions at the moment.

The purpose of this work was to study proangiogenic features in a murine model of colorectal carcinoma cells, obtained from non-hypoxic 3D-cultured CT26 cancer cells spheroids, and to evaluate their functional contribution to hepatic metastasis formation. CT26 spheroids were generated by the hanging-drop method and used prior to hypoxic atmosphere development. Proliferation of cancer cells and recruitment of angiogenic endothelial cells and myofibroblasts were studied in subcutaneous tumors and hepatic metastases generated by subcutaneous and intrasplenic injection of 3D-and monolayer-cultured CT26 cancer cells.

This study demonstrates that culture of CT26 cancer cells as multicellular spheroids leads to the expansion of a LFA-1-expressing cancer cell subpopulation able to further secrete VEGF in response to soluble ICAM-1, via COX-2-dependent mechanism in vitro. In addition, 3D growth-dependent features also endowed cancer cells with an enhanced angiogenic-stimulating potential in vivo, contributing to subcutaneous and metastatic tumor formation. These results suggest that the microenvironment created by the 3D-growth of cancer cells is contributing to the transition from avascular to vascular stages during hepatic colon carcinoma metastasis.

## Materials and methods

### Cell line and maintenance

Murine colon carcinoma cell line (CT26) was obtained from American Tissue Culture Collection (ATCC, Manassas, VA). Cells were cultured in endotoxin-free RPMI 1640 medium supplemented with 10% fetal bovine serum (FBS) and 100 units/ml penicillin and 100 μg/ml streptomycin (all tissue culture reagents were from Sigma-Aldrich, St Louis, MO). Cultures were maintained at 37°C in a humidified atmosphere with 5% CO_2 _and passaged as described previously [11].

### Spheroid culture

CT26 spheroids were generated by the hanging drop method [12]. Five hundred cancer cells suspended in 40 μl of medium (RPMI with 10% FBS and antibiotics) were dispensed into each well of a 48-well culture tray. Trays were then inverted and incubated for 7 days. The number of cancer cells per spheroid was determined by disruption of individual 3D-tissue structures with PBS-EDTA (4 mM, 10 min) and cell counting using a Neubauer chamber. Same procedure was used prior to *in vitro *cancer cell adhesion assays and *in vivo *cancer cell injections in mice.

### Isolation and primary culture of hepatic sinusoidal endothelium (HSE) cells

SyngeneicBalb/c mice (male, 6–8 weeks old) were obtained from Harlan Iberica (Barcelona, Spain). Animal housing, their care, and experimental conditions were conducted in conformity with institutional guidelines that are in compliance with the relevant national and international laws and policies (EEC Council Directive 86/609, OJ L 358. 1, Dec. 12, 1987; and NIH Guide for care and use of laboratory animals. NIH publication 85–23, 1985). HSE cells were separated from these mice, identified, and cultured as previously described [13]. Briefly, hepatic tissue digestion was performed by sequential perfusion of pronase and collagenase, and DNase. Sinusoidal cells were separated in a 17.5% (wt/vol) metrizamide gradient and incubated in glutaraldehyde-treated human albumin-coated dishes for 30 minutes, as a selective adherence step for Kupffer cell depletion. Non-adherent sinusoidal cells were re-plated on type I collagen-coated 24-well plates, at 1 × 10^6 ^cells/ml/well, and 2 hours later were washed. HSE cell purity of resulting adherent sinusoidal cells was around 95% as checked by previously used identification parameters: positive endocytosis (acetylated low density lipoprotein, ovalbumin); negative phagocytosis (1 μm latex particles) and CD45 antigen expression; positive lectin binding-site expression (wheat germ and viscum album agglutinins); and negative vitamin A storage (revealed by 328 nm of UV fluorescence). Cultures of HSE cells were established and maintained in pyrogen-free RPMI (Sigma-Aldrich, St Louis, MO) supplemented with 10% FBS, 100 units/ml penicillin, and 100 μg/ml streptomycin (Sigma-Aldrich, St Louis, MO), at 37°C in a humidified atmosphere with 5% CO_2_.

### Tumor cell adhesion assay to endothelial cells

CT26 cells were labeled with 2',7'-bis-(2-carboxyethyl)-5,6-carboxyfluorescein-acetoxymethylester (BCECF-AM) solution (Invitrogen Co, Carsbad, CA). Next, 2 × 10^5^cells/well CT26 cells grown in monolayer or as spheroids were disrupted with PBS-EDTA (4 mM, 10 min), stained with trypan blue for assessment of cell viability and added to 24-well-plate cultured HSE cells and, 30 minutes later, wells were washed three times with fresh medium. The number of adhering cells was determined using a quantitative method based on a previously described fluorescence measurement system [14].

### Hepatic metastasis

SyngeneicBalb/c mice (male, 6–8 weeks old) were obtained from Harlan Iberica (Barcelona, Spain). Hepatic metastases were produced through the intrasplenic injection into anesthetized mice (0.078 mg/kg ketamine and 6.24 mg/kg xilacin) of 1.8 × 10^5 ^viable CT26 cells (obtained from monolayer- or 3D-spheroid-cultures) suspended in 0.1 ml of Hanks' Balanced salt solution (HBBS). Mice were cervically-dislocated on the 15^th ^day after the injection of cancer cells and livers were removed. Livers were fixed by immersion in Zinc solution for 24 hours at room temperature and, then, paraffin-embedded. A minimum of nine 4-μm thick tissue sections of liver (three groups, separated 1 mm) were stained with H&E. An integrated image analysis system (Olympus Microimage 4.0 capture kit) connected to an Olympus BX51TF microscope was used to quantify the number, average diameter, and position coordinates of metastases. Percentage of liver volume occupied by metastases and metastases density (foci number/100 mm^3^) were also determined [14].

### Immuno-histochemistry

3D-spheroids of various diameters were fixed in 4% paraformaldehide solution and paraffin-embedded, or OCT-embedded and frozen in liquid nitrogen. On the other hand, zinc-fixed livers and primary tumors from subcutaneously-injected mice were also paraffin-embedded. Four micron-thick paraffin sections were obtained from both spheroids and tissue samples and were reacted with 1:50 dilutions of rabbit anti-mouse alpha-smooth muscle actin monoclonal antibody (ASMA) (Zymed, San Francisco, CA), rat anti-mouse CD31 monoclonal antibody (Becton Dickinson, Madrid, Spain), or rat-anti-mouse LFA-1 monoclonal antibody (Acris Antibodies, Hiddenhousen, Germany), or with 1:25 dilutions of rat anti-mouse Ki67 (Dako, Denmark). Their appropriate secondary antibodies were anti-rabbit antibody (dilution 1:100, Dako, Denmark) and rabbit anti-rat antibody (dilution 1:100, Dako, Denmark), respectively. Immuno-labeled cells were detected with an avidin-biotin-phosphatase kit (Vectastain ABC-AP kit, Vector laboratories, Burlingame, CA) according to manufacturer's instructions. Sections were analyzed by quantitative image analysis to determine the number of Ki67-expressing CT26 cells, and the intrametastatic densities of ASMA-expressing cells and CD31-positive capillary cross-sections, as previously described [15,16].

### Cell migration assay

Endothelial cell migration was analyzed with a modified Boyden chamber, as previously described [3]. HSE cells (2.5 × 10^5^) were incubated on 0.01% type I collagen-coated inserts with 8 μm-pores and placed on top of 2 cm^2 ^wells (Becton Dickinson, Madrid, Spain) containing RPMI or conditioned media from either monolayer-or 3D-cultured CT26 cells. After 48 hours, migrated cells were stained with H&E and counted in ×40 high-power fields per membrane.

Conditioned media from CT26 cancer cells were prepared as follows: 5 × 10^6^monolayer-cultured CT26 cancer cells and 143 spheroids on the 7^th ^day of culture (assuming that one single spheroid has 35,000 cells) were incubated in 10 ml of serum-free RPMI 1640 medium, in a 75-cm^2^T-flask, for 12 hours. Supernatants were then collected, 25% fresh serum-free medium supplemented, and 0.22 μm-filtered prior to being used.

### Measurement of VEGF concentration

VEGF concentration was measured using an ELISA kit based on specific murine VEGF monoclonal antibody as suggested by the manufacturer (R&D Systems, Abingdon, UK). Tested supernatants were obtained on the 18^th ^hour of incubation of monolayer- and 3D-spheroid-cultured CT26 cells. For both culture conditions, the concentration of VEGF was expressed as a function of the total number of cultured cells. In some experiments, CT26 cells received 1 μg/ml celecoxib (kindly supplied by Jaime Masferrer, Pfizer, Chesterfield, MO) 30 minutes prior to CT26 treatment with 200 ng/ml recombinant human soluble ICAM-1 (R&D Systems, Abingdon, UK).

### Subcutaneous injection of spheroid- and monolayer-cultured CT26 cells

Balb/c mice received one single subcutaneous injection (using 16 G-syringe) of 0.1 ml serum-free culture medium containing either one CT26 cell spheroid- or an equivalent number of monolayer-cultured CT26 cells (around 35,000 cells for 7-day cultured spheroids). Primary tumors were removed on day 19^th ^after tumor cell injection and fixed in Zinc solution for immuno-histochemical analysis of CD31-expressing neoangiogenic tracts using an integrated image analysis system (Olympus Micro image 4.0 capture kit) connected to an Olympus BX51TF microscope.

### Flow cytometric analysis

CT26 cells were first incubated for 30 min at 4°C with 1 μg/10^6 ^cells of rat anti-mouse LFA-1 antibody (Acris Antibodies, Hiddenhousen, Germany) followed by conjugated alexa-IgG_2a _anti-rat antibody labeling (Invitrogen Co, Carsbad, CA). Cells were then analyzed by flow cytometry using a FACS Vantage SE flow cytometer (Becton Dickinson, Madrid, Spain) by using a wavelength analysis (green: 530 nm) after excitation with 488-nm light. Dead cells (<10%) were excluded from the analysis using Viaprobe (Becton Dickinson, Spain).

### Statistical Analyses

Data were expressed as means ± SD. Statistical analysis was performed by SPPS statistical software for Microsoft Windows, release 6.0 (Professional Statistic, Chicago, IL). Homogeneity of the variance was tested using the Levene test. If the variances were homogeneous, data were analyzed by using one-way ANOVA test with Bonferroni's correction analysis for multiple comparisons when more than two groups were analyzed. For data sets with non-homogeneous variances, ANOVA test with Tamhane'sposthoc was applied. Individual comparisons were made with Student's two-tailed, unpaired t test (program Statview 512; Abacus Concepts, Inc., for Macintosh). The criterion for significance was p < 0.01 for all comparisons.

## Results

### 3D-cultured CT26 cancer cell spheroids

**Figure 1:**
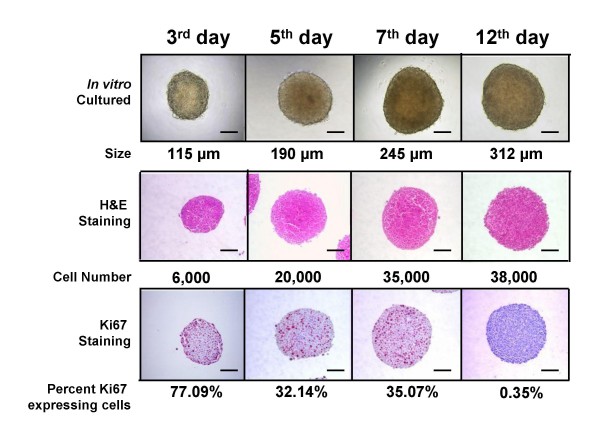
**Growth stages of 3D-cultured CT26 colon cancer spheroid by the hanging-drop method.** Five hundred suspension cancer cells were dispensed into each well of a 48-well culture tray. Trays were then inverted and incubated during 12 days. Spheroids were collected on days 3, 5, 7 and 12 and processed for cell counting, spheroid diameter determination and immunohistochemical detection of Ki67-expressing cells. Scale bar: 100 μm.

**Figure 2:**
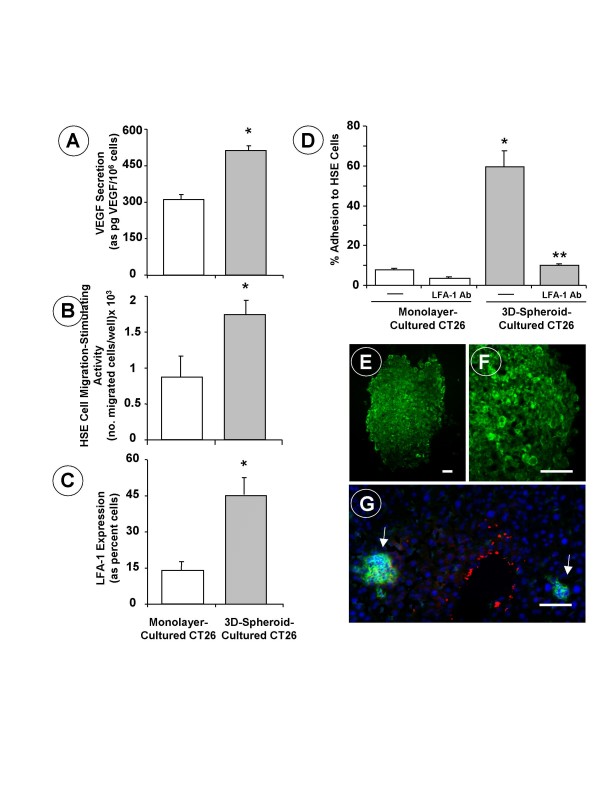
**(A) VEGF secretion by cultured CT26 cells.** Supernatants were obtained on the 18^th ^hour of incubation of CT26 cells, and the concentration of VEGF was determined by ELISA. (B) Hepatic sinusoidal endothelium (HSE) cell migration in response to conditioned media from CT26 cells. Primary cultured HSE cells were incubated for 48 hours with CT26 cell-conditioned media and endothelial cell migration was assayed across type-I collagen-coated inserts. (C) Flow cytometric study on LFA-1 expression. CT26 cells were incubated for 30 minutes at 4°C with 1 μg/10^6 ^cells of rat anti-mouse LFA-1 antibody followed by conjugated alexa-IgG_2a _anti-rat antibody labeling. (D) Adhesion assays of CT26 cells to HSE cells. CT26 cells received 1μg/ml anti-murine LFA-1 antibodies 30 min prior to the adhesion assay. All data from A-to-D studies represent average values ± SD from 3 different experiments (n = 18). Statistical significance: (*) p < 0.01 as compared to monolayer-cultured CT26 cancer cells; (**) p < 0.01 as compared to untreated CT26 cancer cells. (E-F) Inmunofluorescence pictures on LFA-1 expression (green staining) by 3D-spheroid-cultured CT26 cells and (G) a vascular hepatic micrometastases (arrows) on the 7^th ^day after intrasplenic injection of monolayer-cultured CT26 cells. Red staining corresponds to ASMA-expressing fibroblasts around a terminal portal venule and some sinusoids. Scale bar: 20 μm.

Well-rounded compact 3D-spheroids with a homogeneous size distribution were formed in CT26 cancer cell-containing drops suspended from the inverted surface of 48-well microtiter plates. The efficiency level was nearly 100% –i.e., one spheroid per drop and well–, which is in contrast to the low-efficient 3D-growth capability of other cancer cell lines [12]. CT26 spheroids exhibited a highly organized 3D-tissue-like structure where aggregated cancer cells evidenced high proliferation activity until day 7, when the plateau phase of the growth curve was reached by CT26 spheroids, while the percentage of Ki67-expressing cells markedly decreased (Figure 1). The absence of pimonidazole staining in 7-day cultured CT26 spheroids suggests that CT26 spheroids were not affected by hypoxia at this stage of *in vitro *growth (data not shown). However, the concentration of VEGF significantly (P < 0.01) increased in the supernatant of 3D-cultured CT26 cell spheroids compared to the level in monolayer-cultured CT26 cells (Figure 2A). This was particularly visible in the supernatants obtained on the 7^th ^day of spheroidal growth, when VEGF secretion increased by 2-fold, and led to a significant (p < 0.01) increase by 2-fold in the migration of primary cultured HSE cells, as compared to the migration induced by the conditioned medium from an equivalent number of monolayer-cultured cells (Figure 2B).

As shown by flow cytometry, integrin LFA-1-expressing cell fraction also increased by 3-fold in CT26 cancer cells obtained from spheroids (Figure 2C). Immuno-histochemical detection of LFA-1 confirmed that a majority of spheroid-cultured CT26 cells expressed the integrin, both at the peripheral and internal areas of the spheroid (Figure 2E–F). Consistent with this feature, the percentage of CT26 cell adhesion to primary cultured HSE cells significantly (p < 0.01) increased by 6-fold in 3D-spheroid-cultured CT26 cells compared to monolayer-cultured cells; and the addition of 1 μg/ml anti-murine LFA-1 antibody completely abrogated the adhesion of 3D-spheroid-cultured CT26 cells, but not of monolayer-cultured cells, to HSE cells (Figure 2D). Moreover, the majority of smallest avascular CT26 hepatic micrometastases (50–300 μm in diameter) of non-hypoxic character, and that did not extend beyond hepatic lobule limits, were already populated by LFA-1 integrin-expressing CT26 cells (Figure 2G).

### 3D culture-dependent LFA-1 expression accounts for soluble ICAM-1-mediated VEGF secretion by CT26 cells via COX-2-dependent mechanism

**Figure 3:**
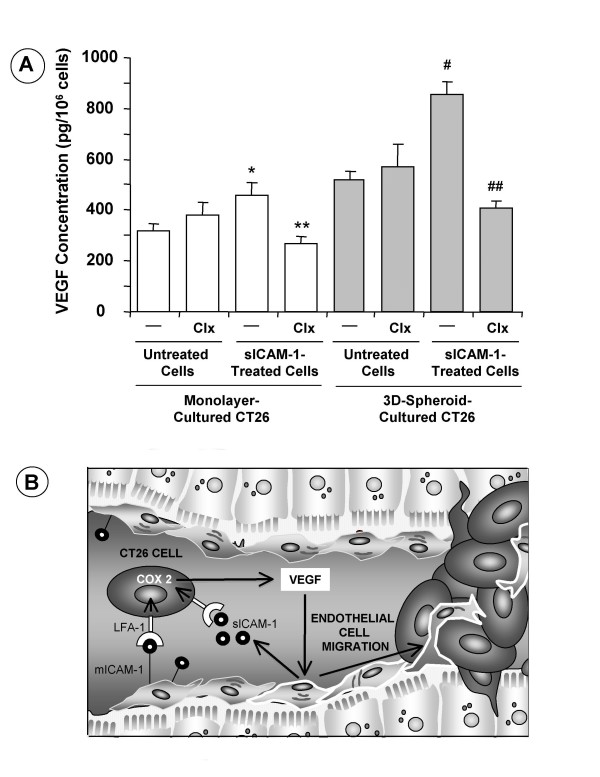
**(A) Effect of COX-2 inhibition on VEGF secretion by recombinant soluble ICAM-1-treated CT26 cells.** In some experiments, CT26 cells received 1 μg/ml of celecoxib 30 min prior to treatment with sICAM-1. VEGF concentration was measured with ELISA in 18-hour supernatants obtained in serum-free culture conditions. Data represent the mean ± SD of three separate experiments, each in six replicates (n = 18). Differences in VEGF secretion with respect to untreated (*) and sICAM-1-treated (**) monolayer-cultured cells, and with respect to untreated (#) and sICAM-treated-(##) 3D-spheroid-cultured CT26-CC cells were statistically significant (p < 0.01) by ANOVA and Bonferroni's post-hoc test. (B) Interaction of tumor LFA-1-expressing CT26 cancer cells with hepatic sinusoidal endothelial cells, via membrane and soluble ICAM-1, induces tumor VEGF overproduction via COX-2 pathway. Next, VEGF induces endothelial cell migration towards a vascular micrometastasis promoting angiogenesis.

In a preliminary study we reported that soluble ICAM-1 increases by two-fold in the supernatant of tumor-activated HSE cells and that, in turn, soluble ICAM-1 increases CT26 cell secretion of VEGF [16]. Herein, both monolayer- and spheroid-cultured CT26 cells received 200 ng/ml recombinant human soluble ICAM-1 for 18 hours and VEGF concentration was determined by ELISA in their supernatants. In these conditions, VEGF secretion potential further increased by 30% and 90% in monolayer- and 3D-spheroid-cultured CT26 cells, respectively, given soluble ICAM-1 (Figure 3A). This remarkable VEGF secretion-stimulating activity of soluble ICAM-1 on 3D-spheroid cultured CT26 cells was consistent with LFA-1-expressing cell number augmentation by 3-fold in 3D-cultured cells compared to monolayer-cultured cells shown in Figure 2A. Because COX-2 contributes to colon carcinoma cell production of VEGF [17], in some experiments, both untreated and soluble ICAM-1-treated monolayer- and 3D-cultured CT26 cells received 1 μg/ml COX-2 inhibitor Celecoxib for 18 hours. VEGF levels did not significantly change in basal condition-cultured CT26 from both 3D-spheroid and monolayer cultures. However, Celecoxib completely abrogated VEGF secretion induced by soluble ICAM-1 on both monolayer- and spheroid-cultured CT26 cells, indicating that VEGF secretion-stimulating activity of soluble ICAM-1 was COX-2-dependent (Figures 3A and 3B).

### 3D-culture-dependent angiogenic potential activation enhances hepatic colonization ability of CT26 cancer cells

**Figure 4:**
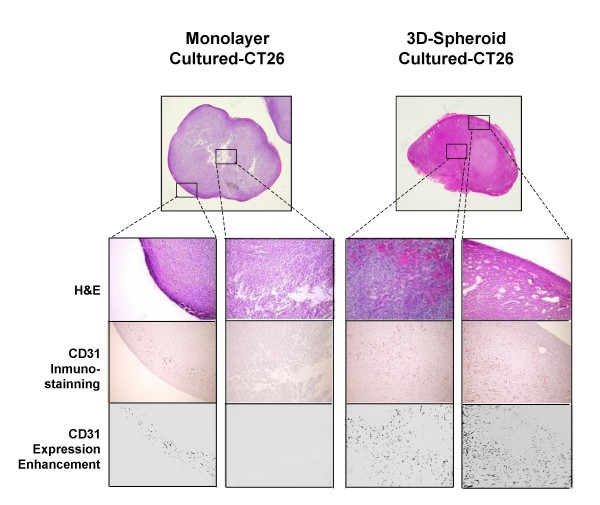
**Angiogenic potential of cancer cells from monolayer and 3D-cultured CT26 cancer cells.** One 3D-spheroid per mouse with a concentration of around 35,000 cells per spheroid was subcutaneously injected in 15 mice (three independent experiments; 5 mice/experiment). The same cancer cell number from monolayer-cultured CT26 was subcutaneously-injected into control mice. Subcutaneous tumors were removed on day 19^th ^after tumor cell injection and processed for CD31 immunostaining. CD31-expression was enhanced by image analysis and CD31-expressing cell density per unit area was determined.

Nineteen days after subcutaneous injection of monolayer- and 3D-cultured CT26 cells, the number of CD31-expressing cells was determined in developed tumors. As shown in Figure 4, a marked recruitment of angiogenic cells occurred at the periphery of subcutaneous tumors generated by CT26 cells derived from both *in vitro *growth conditions. However, only those tumors produced by 3D-cultured CT26 cells were efficiently neovascularized and had angiogenic tracts in the deepest areas of the tumor. Overall, CD31-expressing cell densities per unit area were 1.89 ± 0.56 and 0.66 ± 0.25 in tumors from 3D- and monolayer-cultured CT26 cells, respectively (n = 15 mice from 3 independent experiments having 5 mice per group; differences were statistically significant by the Student's two-tailed, unpaired *t *test, p < 0.01).

**Figure 5:**
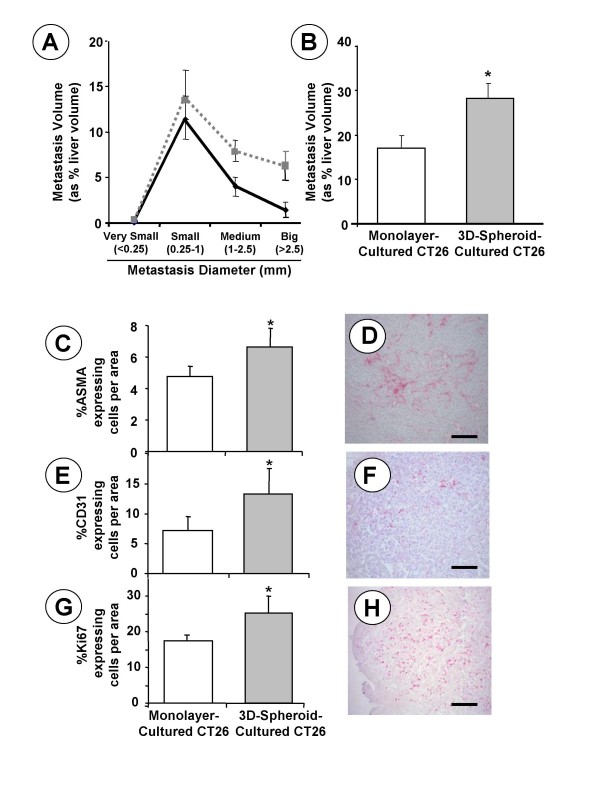
**Monolayer- and 3D-cultured CT26 cancer cells were intrasplenically injected into Balb/c mice (n = 10 per group).** (A) Hepatic metastasis volume fractions are represented according to metastasis size classes (**------**: 3D-spheroid-cultured cells;__________: monolayer-cultured cells). (B) Total hepatic metastasis volumes (as average values) from each mouse group. (C) ASMA-expressing cell number, (E) CD31-expressing endothelial cell number, and (G) Ki67-expressing cancer cell number per unit area of metastasis (0.29 mm^2^) were determined by immunohistochemistry in 3 tissue sections per liver, from 10 livers per group. Differences in average values ± SE were statistically significant with respect to mice injected with monolayer cultured-CT26 cells (p < 0.01) according to the ANOVA and Tamhane's post hoc test. Inmunohistochemical staining of ASMA- (D), CD31- (F) and Ki67- (H) expressing cells in hepatic metastasis. Bar: 200 μm.

Intrasplenic injection of CT26 cancer cells revealed that hepatic metastasis development significantly (*P *< 0.01) increased in mice receiving 3D-spheroid-cultured CT26 cells, as compared to mice given monolayer-cultured CT26 cells (Figure 5A and Figure 5B). This was particularly evident when comparing the metastasis volume indices produced by well-established metastases of medium and big size. Consistent with the proangiogenic activation of spheroid-growing CT26 cancer cells, both endothelial cell (as CD31-expressing cells) and alpha-smooth muscle actin (SMA)-expressing cell numbers significantly (p < 0.01) increased in metastatic nodules produced by 3D-cultured CT26 cells as compared to those developed by monolayer-cultured CT26 cells (Figures 5C–F). As previously described[3], intrametastatic SMA cells were mainly hepatic sinusoidal stellate cell-derived myofibroblasts acting as vascular coverage pericytes of neoangiogenic tumor vessels. Moreover, consistent with the metastatic volume augmentation evidenced in the livers of 3D-spheroid-cultured CT26 cell-injected mice, the average proliferating cell number per unit area of metastatic tissue also significantly increased by 35%, in 3D-spheroid-cultured CT26 cell-injected mice, as detected by immuno-histochemistry using anti-ki67 antibodies (Figures 5G–H).

## Discussion

The results of this study demonstrate that *in vitro *and *in vivo *3D-growth status *per se *activates the proangiogenic phenotype of CT26 cancer cells, prior to hypoxia occurrence. Acquisition of this important feature of the cancer phenotype was evidenced by the significant increase of VEGF secretion when CT26 cancer cells were cultured as 3D-spheroids, and by the remarkable angiogenic tract-formation activity provided by 3D-cultured CT26 cells at both subcutaneous tumors and hepatic metastases. This mechanism was contributed by integrin LFA-1, which significantly increased in both 3D-cultured CT26 cells and non-hypoxic avascular CT26 hepatic micrometastases. In turn, over-expression of this integrin enhanced the adhesion of CT26 to ICAM-1-expressing angiogenic hepatic myofibroblasts and endothelial cells; and endowed CT26 cells with the capability to further increase VEGF secretion, via COX-2, in response to soluble ICAM-1 (Figure 3B), a factor increasing both in the hepatic blood after cancer cell infiltration, and in the peripheral blood of patients affected by numerous cancer types [18,19].

Neoplastic tissues contain a complex spatial organization of growing cancer cells that is missed in traditional monolayer culture systems. Most of structural and functional features of cancer cells are affected by their position coordinates and ambient pressure within tumor tissue, suggesting that biological and therapeutic studies based on two-dimensional cancer cell cultures may lead to inaccurate conclusions that cannot be easily used for translational research and clinical validations. Multicellular spheroids mimic the microenvironment within avascular tumors, and may represent a simple approach to study inducibility of prometastatic factors. Several studies have reported that cancer cell growth as spheroids involves an altered expression profile of cell adhesion molecules [10], and even increased expression of VEGF [20,21]. However, how this is regulated and which functional significance it has *in vivo *are, at the moment, unclear questions.

According to our results, CT26 cells grown either at micrometastatic niches *in vivo*, or as 3D-cancer cell spheroids *in vitro*, markedly increased LFA-1-expressing cell number. Previous studies have already reported that expression of this integrin contributes to hepatic invasion and metastasis of lymphoma, leukemia and breast cancer cells [22,23]; that LFA-1 blockade using specific antibodies can inhibit the hepatic colonization process [24]; and that ICAM-1 deficient mice can prevent post-homing events contributing to the hepatic colonization of T-lymphoma cells [25]. Moreover, HT-29 colon cancer cells grown as spheroids increased CD44 expression [10] and stimulation of this specific cell line by CD44-ligand hyaluronan can induce integrin-mediated adhesion and migration via LFA-1 up-regulation [23]. In the present study, the adhesion of 3D-cultured CT26 cells to primary cultured hepatic sinusoidal endothelial cells increased by 6-fold, and to proangiogenic hepatic stellate cell-derived myofibroblasts, by 2-fold (preliminary data not shown), compared to monolayer-cultured cells. In both cases, this occurred via LFA-1-dependent mechanism as shown by specific anti-LFA-1 blockade. Unlike other microvessels, hepatic sinusoids exhibit elevated base-line expression of ICAM-1 under normal physiological conditions [26,27], suggesting that cancer cell expression ofLFA-1 contributes to retention and seeding of liver-infiltrating colon carcinoma cells. Moreover, activated hepatic myofibroblasts also express ICAM-1 [28] and, therefore, our results suggest that LFA-1 expression may facilitate the functional interaction of cancer cells with ICAM-1-expressing myofibroblasts recruited into smallest micrometastases during early stromagenesis occurring prior to angiogenesis. This stromal-tumor cell interaction may further contribute to VEGF secretion from3D-growing cancer cells within avascular micrometastases.

Our study also shows that recombinant soluble ICAM-1 induced VEGF production from LFA-1-expressing colon cancer cells. This mechanism accounted for 30% of VEGF production from monolayer-cultured CT26 cells, but it augmented VEGF production by 3-fold in 3D-cultured cells. Moreover, there was a strict correlation between LFA-1 expression and VEGF production levels in CT26 colon carcinoma cells activated by soluble ICAM-1. In the liver, metastatic cancer cells that have survived to the cytotoxic environment of the microvasculature start to grow in tight association to hepatic sinusoidal endothelial cells and stellate cell-derived myofibroblasts [3]. Both sinusoidal cell types express and secrete ICAM-1 induced by tumor-derived factors. Soluble ICAM-1 level is also significantly higher in patients with liver metastasis than in those without liver metastasis [18,19]. Therefore, upregulation of LFA-1 expression on cancer cells at this early stage of the hepatic metastasis process may contribute to VEGF production by metastatic cells interacting with liver-derived ICAM-1. However, this mechanism may require the LFA-1-stimulating microenvironment created by the early 3D-growth of cancer cells preceding angiogenesis in the pathogenic cascade of the hepatic metastasis process.

Consistent with this mechanism, it has been reported that both tumor- and host-derived soluble ICAM-1 promote angiogenic activity [29] and support tumor growth [30]. However, our results show for first time that soluble ICAM-1 can directly confer angiogenic-stimulating properties to LFA-1-expressing colon carcinoma cells grown in the hepatic microenvironment. Our results also reveal that the angiogenesis-stimulating potential induced by soluble ICAM-1 on LFA-1-expressing colon carcinoma cells was regulated by cyclooxygenase-2. Upregulation of COX-2 expression has a frequent occurrence in a variety of different tumors including colorectal carcinoma [31,32] and it has been associated to tumor angiogenesis [33]. Because COX-2 accounted for 30% of VEGF from monolayer-cultured CT26 cells, and for 65% of VEGF from 3D-cultured CT26 cells, our results suggest that tumor-derived VEGF is mainly COX-2-dependent during 3D cancer cell growth at the avascular micrometastasis stage (Figure 3B).

Finally, based on a comparative proteomic analysis of cytosolic samples from monolayer- and 3D-cultured CT26 cells we have detected the specific over-expression by 3D-cultured cells of a selected group of biomarker proteins including: 60S acidic ribosomal protein-1, ferritin heavy chain, phosphoglycerate kinase-1, estrogen-related receptor alpha, vimentin and 14-3-3 epsilon (data not shown). Because these proteins have already been associated to mechanisms of cancer progression and tumor angiogenesis, new studies are now in progress to analyze the hepatic pro-metastatic role of this selected ensemble of proteins associated to the 3D-growth of CT26 colorectal carcinoma cells.

## Conclusion

This study demonstrates that culture of CT26 cancer cells as multicellular spheroids leads to the expression of a distinct proangiogenic protein profile, including the specific expansion of a LFA-1-expressing cancer cell subpopulation able to interact with ICAM-1-expressing hepatic endothelial cells and myofibroblasts, and to increase VEGF secretion in response to membrane and soluble ICAM-1, via COX-2-dependent mechanism (Figure 3B). In vivo, CT26 cells also expressed LFA-1 integrin since their earliest 3D-growth of non-hypoxic avascular micrometastasis in the liver, suggesting that 3D-growth-dependent features endowed colorectal cancer cells with an enhanced capability to produce VEGF in response to ICAM-1 provided by tumor-activated hepatic cells. Therefore, the microenvironment created by the 3D-growth of cancer cells may *per se *promote the transition from avascular to vascular stage during hepatic colon carcinoma metastasis.

## Competing interests

The authors declare that they have no competing interests.

## Authors' contributions

MV performed most of *in vitro *and *in vivo *studies and flow cytometry; AJ and CS carried out proteomic studies; AL and FJM performed immuno-histochemical studies; BA and IM contributed to *in vitro *studies; LM participated in its design and coordination, and contributed to *in vitro *and *in vivo *studies; FVV conceived of the study, participated in its design, coordination, and wrote this manuscript. All authors have read and approved the final manuscript.
